# Measuring public health integrity in India: Kerala's COVID-19 undercount as a benchmark for excellence

**DOI:** 10.3389/fpubh.2026.1724793

**Published:** 2026-02-16

**Authors:** Shaiju S. Nazeer, Jithesh Kottur, Jagadeesh Bayry

**Affiliations:** 1 Yenepoya Research Centre, Yenepoya (Deemed to be University), Deralakate/Mangaluru, Karnataka, India; 2 Department of Chemistry, Indian Institute of Space Sciences and Technology, Thiruvananthapuram, Kerala, India; 3 Department of Antiviral Research, Institute of Advanced Virology, Thiruvananthapuram, Kerala, India; 4 Department of Biological Sciences and Engineering, Indian Institute of Technology Palakkad, Palakkad, India

**Keywords:** COVID-19 undercount, excess mortality, Kerala model, mortality surveillance, public health governance

## 1 Introduction

Kerala has long stood apart in India for its exceptional health and human development indicators. With one of the lowest infant mortality rates and highest life expectancy figures in the country, Kerala's health outcomes are comparable to those in many middle- and high-income countries (1). These achievements are rooted in decades of investment in primary health care, education, and social equity, built on a foundation of decentralized governance and strong community participation. These features define the so-called “Kerala Model” of development and also shaped the state's distinctive pandemic response.

Kerala was the first Indian state to report a COVID-19 case in January 2020, yet it rapidly became a global example of effective pandemic containment. Even before confirming its first case, the state-initiated containment measures, drawing on lessons from its 2018 Nipah virus outbreak (2, 3). With early contact tracing, quarantine enforcement, and the deployment of community kitchens and care centers, the state's response was swift, decentralized, and humane. Kerala's pandemic management evolved through multiple waves, including a unique third wave resulting from lower initial seroprevalence and high population mobility. Despite surging case numbers, its health system remained functional, largely due to a tiered care model that prioritized hospital admissions for those in greatest need (4). Kerala's long-standing traditions of public action and participatory democracy initially fuelled a coordinated state–society response. While later waves exposed challenges like bureaucratic tensions, political interference, and public fatigue, the state's transparency, community trust, and resilience largely endured (5).

## 2 India's COVID-19 response

In contrast, India's broader COVID-19 response faced several challenges due to systemic weaknesses. A Lancet editorial described the crisis as a “self-inflicted catastrophe,” citing premature optimism, mass gatherings, and vaccine mismanagement. The devastating second wave, driven by the Delta variant, laid bare the inadequacies of India's public health infrastructure such as oxygen shortages, overwhelmed hospitals, and limited transparency (6). Poor death certification practices, chronic underfunding of public health systems, and weak community engagement were further highlighted. States like Bihar, Uttar Pradesh, and Madhya Pradesh, identified as highly vulnerable, experienced particularly high excess mortality and extreme undercounting (7).

## 3 Undercount as a measure of COVID-19 management integrity

Meanwhile, Kerala's health infrastructure remained resilient. Field hospitals, decentralized care delivery, and surplus oxygen supplies allowed the state to manage surges effectively. Though Kerala reported high case numbers during a third wave, this reflected robust testing and transparent data, often misread as failure rather than a sign of public health integrity (2, 4, 5). Notably, the Economic Survey (2020–21) identified Kerala as the only state in the country that met the recommended norms for healthcare workforce density, whereas all other states failed to reach this benchmark (8).

Excess mortality is measured as the difference between observed all-cause deaths and expected historical baselines. Its ratio to officially reported COVID-19 fatalities, known as the undercount ratio, serves as a reliable indicator of the accuracy and integrity of mortality reporting during the pandemic. Unlike reported case or death counts, excess mortality provides a more reliable measure of the pandemic's true impact, as it accounts for inconsistencies in testing, cause-of-death attribution, and political interference in reporting (9).

**Figure 1:**
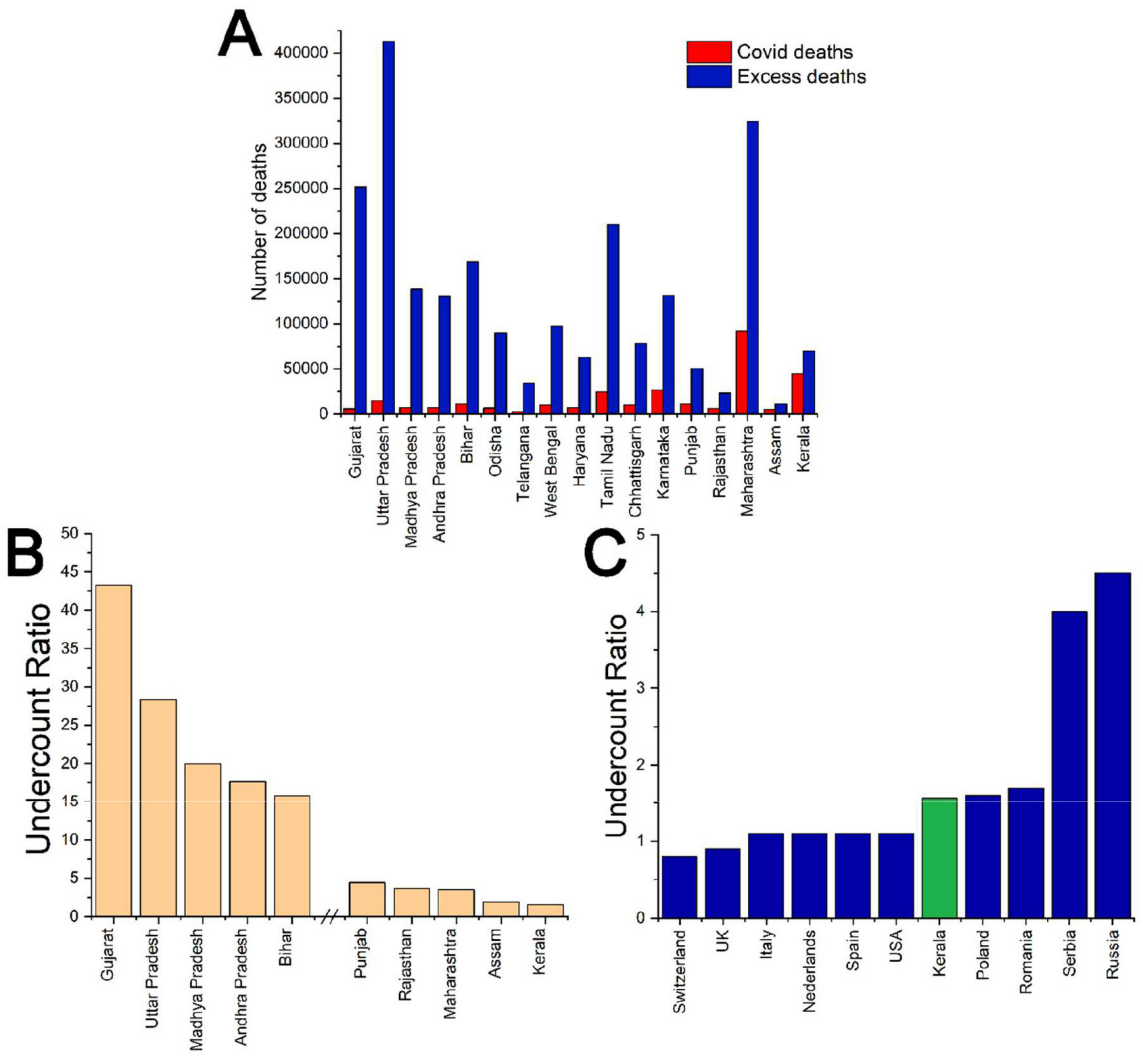
**(A)** Comparison of officially reported COVID-19 deaths (red) and estimated excess deaths (blue) across selected (total death >1.5 lakhs in 2019) Indian states in 2021. **(B)** Undercount ratios of COVID-19 mortality in selected Indian states, showing the five highest and five lowest estimates. **(C)** Comparison of undercount ratios of COVID-19 deaths between Kerala and selected countries in Europe and North America (9) [Excess deaths were estimated using (10), and cumulative COVID-19 deaths as of Dec 31, 2021, were obtained from (11)].

India's Civil Registration System data for 2021 revealed 10.2 million deaths, far exceeding historical baselines (10). Figure 1 starkly illustrates the reporting gaps: states like Gujarat (undercount ratio of 43.3), Uttar Pradesh (28.4), and Madhya Pradesh (19) reported only a fraction of their actual COVID-19 fatalities. In sharp contrast, Kerala's undercount ratio was just 1.57, meaning that for every confirmed COVID death, fewer than two went unreported. This level of accuracy aligns closely with developed countries such as the United States (1.1), the United Kingdom (0.9), and Italy (1.1), underscoring Kerala's relative transparency and robust data integrity (9, 11).

An important consideration in interpreting Kerala's comparatively better COVID-19 outcomes is the role of economic factors. While economic capacity can influence health system preparedness, Kerala is not the richest state in India by conventional economic measures. For the financial year 2024–25, Kerala ranked seventh in per capita Net State Domestic Product, eleventh in Gross State Domestic Product, and ninth in Net State Domestic Product, placing it behind several economically stronger states (12). Despite this, Kerala has recently become the first Indian state to eradicate extreme poverty, an achievement not yet realized in many higher-income regions (13). This highlights the importance of equitable social investment, rather than absolute economic wealth, in shaping population health outcomes.

Kerala's COVID-19 performance is better explained by long-term public health policies and governance choices than by economic affluence alone. Sustained investment in primary health care, a strong public sector hospital network, and the mobilization of grassroots health workers enabled early detection, timely reporting, effective contact tracing, and isolation. High literacy and decentralized governance further supported risk communication and local accountability. These factors distinguish Kerala from several economically stronger states that nonetheless experienced health system strain, substantial underreporting, and high excess mortality, underscoring that governance quality and social investment matter more than income rankings alone.

Kerala's extensive medical certification of COVID-19 deaths, strong death registration systems, and willingness to revise mortality figures in response to judicial and expert recommendations, reflect a clear commitment to data accuracy. This reflects not merely administrative competence, but the operationalization of ethical standards within public health governance. While states like Maharashtra and Tamil Nadu also had significant excess deaths, their undercount ratios remained above 3 and 8, respectively. Kerala alone mirrored the transparent practices of developed nations, reflecting its structural strengths and public accountability mechanisms.

Importantly, Kerala's reporting integrity challenges the myth that high case or death counts reflect governance failure. On the contrary, visibility becomes a virtue when combined with responsive health services and informed citizens. Kerala's undercount ratio thus becomes not just a statistic, but a benchmark for public health integrity.

## 4 Conclusion

As India and Southeast Asia confront with the long-term consequences of the COVID-19 pandemic, future health governance must be grounded in transparency, public trust, and institutional accountability. Timely reporting of cases is crucial and far preferable to concealing or failing to disclose a disease. Kerala's experience, rooted in strong local governance, community engagement, and a commitment to data integrity, offers a compelling model. While imperfect, it illustrates that resilience is shaped not only by capacity but also by values. Kerala's case thus underscores the importance of robust civil registration systems, independent media, and a culture of accountability in shaping pandemic outcomes.
